# Single-Cell-Resolution Fate Mapping Reveals Embryonic Venous Origins of Fenestrated Hindbrain Choroid Plexus Vasculature

**DOI:** 10.1523/JNEUROSCI.2204-25.2026

**Published:** 2026-03-12

**Authors:** Nathanael J. Lee, Fatma Z. Bozdag, Jun Xiong Leong, Sweta Parab, Amanda E. Lam, Vani Thakur, Ryota L. Matsuoka

**Affiliations:** ^1^Department of Neurosciences, Lerner Research Institute, Cleveland Clinic, Cleveland, Ohio 44195; ^2^Department of Molecular Medicine, Cleveland Clinic Lerner College of Medicine, Case Western Reserve University, Cleveland, Ohio 44195

**Keywords:** blood–brain barrier, brain angiogenesis, choroid plexus, endothelial lineages, fenestrated vessels, vascular heterogeneity

## Abstract

In the brain, endothelial cell (EC) subtypes characterized by blood–brain barrier (BBB) properties or fenestrated pores form essential brain–blood interfaces and exhibit markedly distinct permeability. The choroid plexus (CP) establishes fenestrated vasculature lacking the BBB to efficiently regulate cerebrospinal fluid balance, yet its developmental origins and mechanisms remain poorly defined. Using single-cell-resolution fate mapping in zebrafish, we identify here two venous sources that give rise to the hindbrain myelencephalic CP (mCP) vasculature. RNAscope and BAC transgenic analyses reveal highly abundant and persistent expression of the venous marker *flt4* in these EC lineages, supporting their identities. Unexpectedly, we find that these venous origins of the mCP vasculature also contribute ECs to diverse cranial vessels, including those that maintain low *flt4* expression and later acquire BBB characteristics. Functionally, *flt4* null and cytoplasmic-domain-deletion mutants exacerbate mCP vascularization defects when combined with *vegfr2* signaling deficiency, without disrupting neighboring BBB-type vessels. Pharmacological data support this corequirement of Flt4 and Vegfr2 signaling in mCP vascularization and further suggest that the PI3K and ERK pathways are necessary for this process. Together, these findings reveal embryonic venous lineages and molecular pathways required for hindbrain CP vascularization and imply that Flt4 signaling contributes to the angiogenic separation of CP- and BBB-associated capillaries originating from shared embryonic domains.

## Significance Statement

The choroid plexus forms permeable vasculature devoid of the blood–brain barrier. This vasculature is vital for maintaining brain homeostasis by facilitating molecular exchange between blood and cerebrospinal fluid, yet its developmental mechanisms remain poorly understood. In this study, we investigated embryonic endothelial origins and molecular pathways underlying hindbrain choroid plexus vasculature. Our fate-mapping results, together with genetic and pharmacological data, show that choroid plexus vasculature arises exclusively from embryonic veins expressing the venous marker *flt4*, in part dependent on its receptor function. Further expression analyses suggest that differential cerebrovascular *flt4* expression guides the developmental separation of choroid plexus vasculature and barrier-associated vessels. These findings advance our understanding of choroid plexus vascularization and provide new insights into heterogeneous cerebrovascular development.

## Introduction

Brain vasculature exhibits prominent permeability differences across regions. This difference was first observed in the early 1900s, when studies showed that intravenous injections of trypan blue strongly stained the choroid plexus (CP), but not the brain parenchyma (Goldmann, 1909, 1913). Subsequent studies addressed the evolutionary conservation of this phenomenon in various species and characterized cerebrovascular barrier and non-barrier properties at anatomical and functional levels (Saunders et al., 2014; O'Brown et al., 2018). Current evidence suggests that cerebrovascular endothelial cells (ECs) play a central role in regulating brain region-specific vessel permeability by exhibiting phenotypic diversity (Vanlandewijck et al., 2018; Kalucka et al., 2020). This diversity is particularly evident at the capillary level, where ECs establish the semipermeable BBB in most regions but form permeable fenestrations lacking the BBB in the CPs and circumventricular organs (CVOs; Miyata, 2015; Profaci et al., 2020). These BBB and fenestrated EC subtypes are critical for versatile neural functions and brain homeostasis, yet the developmental processes leading to this divergent cerebrovascular network remain unclear.

Like other peripheral organs, brain vascularization relies largely on angiogenesis. Blood vessel invasion into the brain parenchyma begins with sprouting angiogenesis from the perineural vascular plexus (Strong, 1964; Walchli et al., 2015). This process requires tip cell specialization dependent on Wnt7/β-catenin signaling and its downstream effector, Mmp25 (Vanhollebeke et al., 2015; Schevenels et al., 2024), where Gpr124 and Reck serve as receptor cofactors crucial for β-catenin activation (Kuhnert et al., 2010; Vanhollebeke et al., 2015; Cho et al., 2017). Subsequently, endothelial β-catenin drives gene transcription critical for establishing BBB properties (Liebner et al., 2008; Benz et al., 2019; Wang et al., 2019). These angiogenic and BBB induction mechanisms are evolutionarily conserved between mammals and zebrafish (O'Brown et al., 2018; Matsuoka et al., 2022).

Conversely, much less is known about the developmental mechanisms governing fenestrated vascular formation in the CPs and CVOs. Physiologically, these organs regulate neural development and homeostasis by producing growth factors, neuropeptides, and hormones that are partly secreted into the bloodstream through fenestrated vessels (Siso et al., 2010; Saunders et al., 2023). In addition, the CP has recently received increasing attention in the context of central nervous system (CNS) pathologies, because of its vital roles in forming the blood–cerebrospinal fluid (CSF) barrier, serving as a hub for immune surveillance, and maintaining fluid homeostasis through the production and clearance of CSF (Ghersi-Egea et al., 2018; Courtney et al., 2025). Similarly, the association of CVOs with neurological, endocrine, and autoimmune disorders has been implicated, due to their crucial roles in mediating neuroendocrine regulation, fluid interoception, and neural immunity (Miyata, 2015; Kovacs et al., 2025). Importantly, fenestrated vasculature underlies many of these functions in the CPs and CVOs as a shared structural feature; however, their vascularization processes remain poorly characterized.

Our recent studies suggest that Vegfa and Vegfc/d ligands are co-required for angiogenesis leading to CP and CVO vascularization in zebrafish, with this requirement fulfilled locally through interactions between growing ECs and specialized cell types residing in these organs (Parab et al., 2021; Parab et al., 2023; Lee and Matsuoka, 2025). Interestingly, despite the severe loss of CP vasculature in the absence of these Vegfs, the formation of neighboring BBB vessels remains largely unaffected (Parab et al., 2021). This striking vessel specificity raises the question of what molecular mechanisms determine ECs’ differential responsiveness to specific angiogenic cues in the local microenvironment.

Here, we investigated the mechanisms underlying this vessel specificity by examining embryonic EC lineages and molecular pathways that drive CP vascularization. Combining high-resolution fate mapping and expression analysis with genetic and pharmacological manipulations, we identify embryonic venous lineages and Vegf receptor (Vegfr) pathways crucial for CP vascularization. During these characterizations, we unexpectedly find embryonic vascular domains from which ECs migrate divergently to form fenestrated CP and BBB-type blood vessels. Our further analyses indicate that Flt4 functions as a specific angiogenic receptor guiding ECs toward CP vascularization.

## Materials and Methods

### Zebrafish husbandry and strains

All zebrafish husbandry was performed under standard conditions in accordance with institutional and national ethical and animal welfare guidelines. All zebrafish work was approved by the Cleveland Clinic’s Institutional Animal Care and Use Committee under the protocol number 00003285. The following lines were used in this study: *Tg(kdrl:EGFP)^s843^* (Jin et al., 2005); *Tg(kdrl:Has.HRAS-mcherry)^s896^* (Chi et al., 2008), abbreviated *Tg(kdrl:ras-mCherry)*; *Tg(kdrl:TagBFP)^mu293^* (Matsuoka et al., 2016); *TgBAC(flt4:Citrine)^hu7135^* (van Impel et al., 2014); *KI(etv2−2A-Gal4)^ci32Gt^* (Chestnut and Sumanas, 2020); *Tg(UAS:Kaede)^rk8^* (Hatta et al., 2006); *Et(cp:EGFP)^sj2^* (Henson et al., 2014); *Tg(glut1b:mCherry)^sj1^* (Umans et al., 2017), abbreviated *Tg(glut1:mCherry)*; *Tg(plvapb:EGFP)^sj3^* (Umans et al., 2017), abbreviated *Tg(plvap:EGFP)*; *TgBAC(vegfab:gal4ff)^bns273^* (Mullapudi et al., 2019); *TgBAC(vegfc:gal4ff)^bns270^* (Parab et al., 2021); *TgBAC(vegfd:gal4ff)^lri95^* (Parab et al., 2021); *Tg(UAS:EGFP-CAAX)^m1230^* (Fernandes et al., 2012); *vegfc^hu6410^* (Helker et al., 2013); *flt1^bns29^* (Matsuoka et al., 2016); *kdrl^um19^* (Covassin et al., 2009); *kdr^bns32^* (Mullapudi et al., 2019); *flt4^um131^* (Kok et al., 2015); and *flt4^um200^* (Shin et al., 2016). Adult fish were maintained on a standard 14/10 h light/dark daily cycle. Fish embryos and larvae were raised at 28.5°C, during which stages sex could not be determined. To prevent skin pigmentation, 0.003% phenylthiourea (PTU) was used beginning at 10–12 hpf for imaging. Fish larvae analyzed at 10 dpf were transferred to a tank containing ∼250 ml water supplemented with 0.003% PTU (up to 25 larvae/tank) and fed with Larval AP100 (<50 microns dry diet, Zeigler) starting at 5 dpf.

### Genotyping of mutants

Genotyping of *flt1^bns29^* mutant fish was performed by high-resolution melt analysis (HRMA) of PCR products as described previously (Matsuoka et al., 2016). Genotyping of *kdrl^um19^*, *kdr^bns32^*, and *flt4^um131^* mutant fish was performed by HRMA of PCR products using the following primers:

*kdrl um19* forward: 5′ – TGCTTCCTGATGGAGATACACACC – 3′
*kdrl um19* reverse 5′ – TGCAAATGAGTGTGAGTGTCCCAC – 3′
*kdr bns32* forward: 5′ – GACCTCACCCTGAGTCCACA – 3′
*kdr bns32* reverse: 5′ – GCGGTGCAGTTGAGTATGAG – 3′
*flt4 um131* forward: 5′ – GACCATCTTCATAACAGACTCTG – 3′
*flt4 um131* reverse: 5′ – GGATCTGAAACCAGACATGGTAC – 3′

Genotyping of *flt4^um200^* mutant fish was performed by PCR using the following primers:

*flt4 um200* forward: 5′ – GAAATCCCGTTCAATGTGTCTCAG – 3′
*flt4 um200* reverse: 5′ – CATTTCCATAGGAAGTTCCTCAAAG – 3′
*flt4 um200* forward: 5′ – acgtagccttatataactaagccc – 3′

The PCR reaction yielded products of 459 bp for the WT allele and 144 bp for the mutant allele.

### High-resolution melt analysis (HRMA)

A CFX96 Touch Real-Time PCR Detection System (Bio-Rad) was used for the PCR reactions and subsequent HRMA. Precision melt supermix for high-resolution melt analysis (Bio-Rad) was used in these experiments. PCR reaction protocols were as follows: 95°C for 2 min, 46 cycles of 95°C for 10 s, and 60°C for 30 s. Following the PCR, a high-resolution melt curve was generated by collecting EvaGreen fluorescence data in the 65–95°C range. The analyses were performed on normalized derivative plots.

### Generation of F0 knock-outs (crispants)

Dual-guide ribonucleoprotein complexes (dgRNPs) consisting of CRISPR RNA (crRNA), trans-activating RNA (tracrRNA), and Cas9 protein were prepared as previously described (Quick et al., 2021; Lee and Matsuoka, 2026). The following sequences of *vegfab* and *vegfc* crRNAs were used:

*vegfab* crRNA1: 5′ – AGTGCCTACATACCCAGAGA – 3′
*vegfab* crRNA2: 5′ – TTGTTATACACCTCCATGAA – 3′
*vegfab* crRNA3: 5′ – GAAGCGTCACAATAAATAAC – 3′
*vegfc* crRNA1: 5′ – CGTGACTTGACTCGAAAGCA – 3′
*vegfc* crRNA2: 5′ – CTGAACGCAACTGCTCCACC – 3′
*vegfc* crRNA3: 5′ – AAGTAAGCGCGACCCCATCT – 3′

Injection cocktails (4 μl) containing both *vegfab* and *vegfc* dgRNPs were prepared as follows: 0.4 μl 25 μM *vegfab* crRNA1:tracrRNA, 0.4 μl 25 μM *vegfab* crRNA2:tracrRNA, 0.4 μl 25 μM *vegfab* crRNA3:tracrRNA, 0.4 μl 25 μM *vegfc* crRNA1:tracrRNA, 0.4 μl 25 μM *vegfc* crRNA2:tracrRNA, 0.4 μl 25 μM *vegfc* crRNA3:tracrRNA, 1.2 μl 50 μM Cas9 protein stock, and 0.4 μl 0.5% phenol red solution (#P0290, Sigma). Injection cocktails (4 μl) containing only *vegfab* dgRNPs were prepared as follows: 0.4 μl 25 μM *vegfab* crRNA1:tracrRNA, 0.4 μl 25 μM *vegfab* crRNA2:tracrRNA, 0.4 μl 25 μM *vegfab* crRNA3:tracrRNA, 1.2 μl 25 μM Cas9 protein stock, 1.2 μl H_2_O, and 0.4 μl 0.5% phenol red solution. Injection cocktails were freshly prepared on the day of injection, and ∼2 nl of the cocktails was injected into the cytoplasm of one-cell stage embryos.

### Immunohistochemistry

Immunohistochemistry was performed by following standard immunostaining procedures as described previously (Parab et al., 2021), except for phosphorylated ERK (pERK) immunostaining that is detailed below. Zebrafish embryos and larvae were fixed in pH adjusted (pH 7.0), 4% paraformaldehyde (PFA)/phosphate-buffered saline (PBS) overnight at 4°C and dehydrated through immersion in methanol serial dilutions (50%, 75%, then 100% methanol three times, 10 min each) at room temperature (RT). Dehydrated samples were stored in 100% methanol at −20°C until use. Samples were rehydrated through immersion in methanol serial dilutions (50%, then 25% methanol, 10 min each) at RT and washed briefly three times in 1% PBST (1% Triton X-100 in 0.1 M PBS) followed by permeabilization in 10 µg/ml Proteinase K in 1% PBST at RT for 15 min. Samples were blocked at RT for 2–4 h in the blocking solution containing 0.5% bovine serum albumin in 1% PBST prior to primary antibody incubation.

Antigen retrieval of pERK immunostaining was performed as follows. After rehydration, samples were briefly equilibrated in the antigen retrieval buffer (150 mM Tris-HCl, pH 9.0) at RT for 5 min. Samples were then heated in this buffer at 70°C for 15 min. Samples were washed three times in 1% PBST at RT for 5 min each and permeabilized in 10 µg/ml Proteinase K in 1% PBST at RT for 15 min. Samples were then processed in the same manner as described above.

The following primary antibodies were used: chicken anti-GFP (GFP-1010, Aves Labs, 1:1,000), rabbit anti-DsRed (#632496, Clontech, 1:300), and rabbit anti- Phospho-ERK1/2 (#4370S, Cell Signaling, 1:250). After primary antibody incubation at 4°C overnight, all samples were washed well, incubated with secondary antibodies, and processed for imaging, as described previously (Parab et al., 2021).

### RNAscope in situ hybridization

RNAscope assays were performed on *Tg(kdrl:EGFP)^s843^* embryos and larvae collected at 43 hpf, 55 hpf, 75 hpf, and 10 dpf. Samples were fixed, dehydrated, and rehydrated as detailed in the immunohistochemistry procedure. Permeabilization was performed using Protease Plus (#322331; Advanced Cell Diagnostics, ACD) at 40°C in the HybEZ II hybridization system (#321711, ACD) for 2.5, 3.5, 4, and 10 min for 43 hpf, 55 hpf, 75 hpf, and 10 dpf samples, respectively. After permeabilization, samples were washed three times in 1% PBST at RT and transferred to 2 ml Eppendorf tubes (10–12 fish per tube). Negative control and gene-specific probes were designed and synthesized by ACD and prepared according to their instructions. To optimize this wholemount protocol for zebrafish samples, a probe targeting the bacterial gene *dapB* (#320871, 1×) was used as a negative control and confirmed to produce no detectable background signals. Probes specific to *flt1* (#815251-C3, 50×), *kdrl* (#416611-C2, 50×), and *flt4* (#1205121-C2, 50×) were freshly diluted in probe diluent (#300041, ACD) for each assay. A *kdr*-specific probe (#1318021-C1, 1×) was used without dilution. Hybridization with each probe was performed at 40°C for 2 h. During this incubation, 1× wash buffer was diluted from the 50× stock (#310091, ACD) in H_2_O and prewarmed.

Following hybridization, signal detection was carried out using the RNAscope Multiplex Fluorescent Detection Kit v2 (#323110, ACD). Samples were washed three times in prewarmed wash buffer at 40°C for 10 min each. After this step, prewarmed amplification reagents (Amp1, Amp2, Amp3) were sequentially applied at 40°C for 30, 30, and 15 min, respectively, with two intervening washes in wash buffer at RT for 5 min each. After incubation with Amp3, samples were washed two times in wash buffer at RT for 5 min each and kept in 5× SSC (750 mM NaCl, 75 mM sodium citrate, pH 7.0) at RT overnight.

The following day, after washing once in wash buffer for 5 min at RT, samples were incubated with prewarmed appropriate HRP-conjugated reagents (HRP-C3, HRP-C2, or HRP-C1) at 40°C for 15 min followed by two washes in wash buffer at RT for 5 min. Fluorescent signal was developed using TSA Vivid Fluorophore 650 (#323273, ACD) diluted at 1:750 in Multiplex TSA buffer (#322810, ACD) at 40°C for 30 min. After this step, samples were protected from prolonged light exposure to preserve fluorescence. After washing twice in wash buffer at RT for 5 min each, samples were treated with prewarmed Multiplex FL v2 HRP Blocker (#323107, ACD) at 40°C for 15 min, washed once in 1% PBST at RT, and incubated with anti-GFP antibody described earlier at RT for 4–6 h. After three washes in 1% PBST at RT for 20 min each, samples were incubated with a secondary antibody at 4°C overnight. After six washes in 1% PBST at RT for 15 min each, samples were processed for imaging.

### Quantification of RNAscope assays

Gene expression in the specified blood vessels was quantified using high-resolution images acquired from RNAscope assays. For 43 hpf samples, a 20 μm × 5 μm area was selected within PCeV tip cells and the PMBC, and the number of mRNA puncta overlapping with *Tg(kdrl:EGFP)*-positive ECs was manually counted. For 55 and 75 hpf samples, a 20 μm × 20 μm area was selected within the DLV, PCeV, MsV, and MCeV, and mRNA puncta overlapping with *Tg(kdrl:EGFP)*-positive ECs were manually counted. The specific locations used for this quantification are indicated in the vascular diagram shown in Figure 6*B*. For 10 dpf larvae, a 50 μm × 50 μm area was quantified at locations similar to those used for 55 and 75 hpf embryos/larvae. For probes with some background signals, only the bright signals were counted. For most probes, maximum projection *z*-stack images were used for signal counting, as these probes displayed highly specific vascular signals. For some probes with strong non-blood vessel signals, *z*-stack images were carefully checked stack-by-stack to count only signals overlapping with *Tg(kdrl:EGFP)*-positive ECs. The quantified data for all probes using this method well reflect our visual observations of the gene expression patterns.

### Photoconversion

Photoconversion experiments were performed in embryos carrying the *KI(etv2-2A-Gal4)^ci32Gt^* and *Tg(UAS:Kaede)^rk8^* transgenes using a Leica SP8 confocal microscope as follows. A *z*-stack confocal image was first captured for each embryo to select target cells for photoconversion. Using the bleach point setting in Leica Application Suite X (LAS X) software, a region of interest (ROI) was designated for each target cell. Either single or multiple cells were targeted per embryo. Photoconversion of Kaede from green to red fluorescence was achieved by applying 405 nm laser illumination (1% power) for 1 s. If photoconversion appeared incomplete, an additional illumination for 1 s was applied. After successful photoconversion, bleach point setting was deactivated, and post-photoconversion images were captured. Photoconversion in *vegfab;vegfc* crispant embryos was performed in the same manner, except that these embryos were injected at the one-cell stage with a cocktail containing both *vegfab* and *vegfc* dgRNPs.

For longitudinal cell tracking, photoconverted embryos were carefully rescued from agarose gels using a glass pipette, transferred to 24-well plates containing PTU egg water, and maintained at 28.5°C. Images were captured ∼24 h and/or 48 h later to assess migration locations of photoconverted cells. For time-lapse imaging involving photoconversion, embryos were imaged immediately after photoconversion in a temperature-controlled chamber maintained at 28.5°C. Images were acquired at 10 min intervals from ∼45 to 55 hpf to monitor dynamic behaviors of photoconverted cells.

### Quantification of photoconverted cell locations originating from the PCeV, PHS, ACV, and PHBC

To assess the migration locations of ECs photoconverted at 42 hpf in the PCeV tip position, PHS, ACV, or PHBC, their positions were examined at 64 hpf. For quantification, migration positions were determined based on the locations of photoconverted cell bodies and categorized into five locations: mCP, dorsal PCeV, ventral PCeV, PHS, and ACV. For detailed vessel categorization used for this quantification, please refer to the schematic diagram shown in Figure S1G.

### Quantification of photoconverted cell locations originating from the PPAJ

To assess the migration locations of PPAJ cells photoconverted at 30 hpf, their positions were examined at 50 hpf. For quantification, migration positions were determined based on the locations of photoconverted cell bodies and categorized into four locations: mCP/PCeV, PPAJ, PHS, and PHBC/CtA.

### Quantification of photoconverted cell locations originating from the MCeV

To quantify the migration locations of photoconverted MCeV cells, their initial positions at 43 hpf were measured relative to the midline using a defined ROI. These positions were categorized based on their distances from the midline on both hemispheres, as shown in Figure 3*R*. Cells located within 50 µm from the midline were assigned to the 1st position, while those located between 50 and 100 µm were assigned to the 2nd position. When multiple targeted cells within an embryo were distributed across both positions, data from these embryos were excluded from the graphs shown in Figure 3*R* but were included in the graph presented in Figure 4*P*. For quantification, migration positions were determined based on the locations of photoconverted cell bodies at 67 hpf and categorized into five locations: DLV/PCeV (mCP vessels), MCtA, MsV, MsV–DLV junctions, and MCeV.

### Confocal and stereo microscopy

Fluorescence imaging of live and fixed zebrafish samples was conducted using a Leica TCS SP8 confocal laser scanning microscope. Fish embryos and larvae were anaesthetized with a low dose of tricaine, embedded in 1% low melt agarose in glass-bottom Petri dishes (MatTek), and imaged using a 25× water immersion objective lens. Image acquisition and analysis were conducted using LAS X software (version 3.7.0.20979). Bright-field images of anesthetized fish were captured using a Nikon SMZ-18 stereomicroscope. Image acquisition and analysis for bright-field imaging were carried out using NIS-Elements BR Imaging software (version 5.10.01).

### Time-lapse cell tracking

*Tg(kdrl:EGFP)^s843^* embryos were used for time-lapse live imaging between 34 and 48 hpf. Embryos mounted in a glass-bottom dish were placed in a temperature-controlled chamber (Okolab) maintained at 28.5°C. Images were acquired every 8 min for each embryo to allow time-lapse recording of vascular growth in individual embryos over a 14.5 h period. Image-Pro 11 software (Media Cybernetics) was used to analyze the acquired time-lapse movies and track targeted cell behaviors in each time-lapse frame. Orientation plots for tracked cells were generated using the DiPer method as previously described (Gorelik and Gautreau, 2014). Specifically, the Plot_At_Origin program was used to visualize each cell’s displacement trajectory by plotting all tracks with their starting positions aligned to position (0, 0) in the XY plane.

### 3D reconstruction of embryonic cranial vasculature

*KI(etv2-2A-Gal4)^ci32Gt^;Tg(UAS:Kaede)^rk8^* embryos were used to collect *z*-stack confocal images at 1 µm intervals with or without photoconversion. The acquired *z*-plane images were subsequently reconstructed in 3D using the Imaris software (Bitplane).

### Pharmacological treatment

Embryos carrying the *Tg(kdrl:EGFP)^s843^* transgene were used for pharmacological experiments. Treatments were conducted from 34 to 72 hpf with the following inhibitors: SL327 (#S4069, Sigma), LY294002 (#L9908, Sigma), U73122 (#U6756, Sigma), SKLB1002 (#SML1164, Sigma), and MAZ51 (#676492, Sigma). At 54 hpf, each drug solution was replaced with a fresh preparation to ensure consistent efficacy of the chemicals. Following our previous protocol (Matsuoka et al., 2017), all chemicals were initially prepared as 100× stock solutions dissolved in 100% DMSO and diluted 1:100 in egg water immediately before use.

### Quantification of DLV, PCeV, MsV, and MCeV formation

Quantification was performed using fish carrying the *Tg(kdrl:EGFP)^s843^* reporter. Fish larvae at the indicated developmental stages were analyzed for the presence or absence of the DLV. In larvae where the DLV was present, the lengths of the DLV were measured by drawing a ROI using the polyline tool in LAS X software. To assess the bilateral formation of the PCeV, MsV, and MCeV, the following scoring criteria were applied: (1) Score 2—both bilateral vessels are fully formed; (2) Score 1.5—one vessel is fully formed, while the other is partially formed; (3) Score 1—both vessels are partially formed, or one vessel is fully formed while the other is absent; (4) Score 0.5—one vessel is partially formed, while the other is absent; and (5) Score 0—both bilateral vessels are absent.

### Statistical analysis

Statistical differences in mean values among multiple groups were determined using a one-way analysis of variance (ANOVA) followed by Tukey's multiple-comparison test. Fisher’s exact test was applied to assess significance when comparing the degree of observed phenotypic penetrance. Statistical analyses were conducted using GraphPad Prism 8.1.1. The criterion for statistical significance was set at *p* < 0.05. Error bars represent SEM.

## Results

### A photoconversion-based approach enables single-cell-resolution tracking of embryonic ECs that give rise to fenestrated mCP vasculature

**Figure 1.**
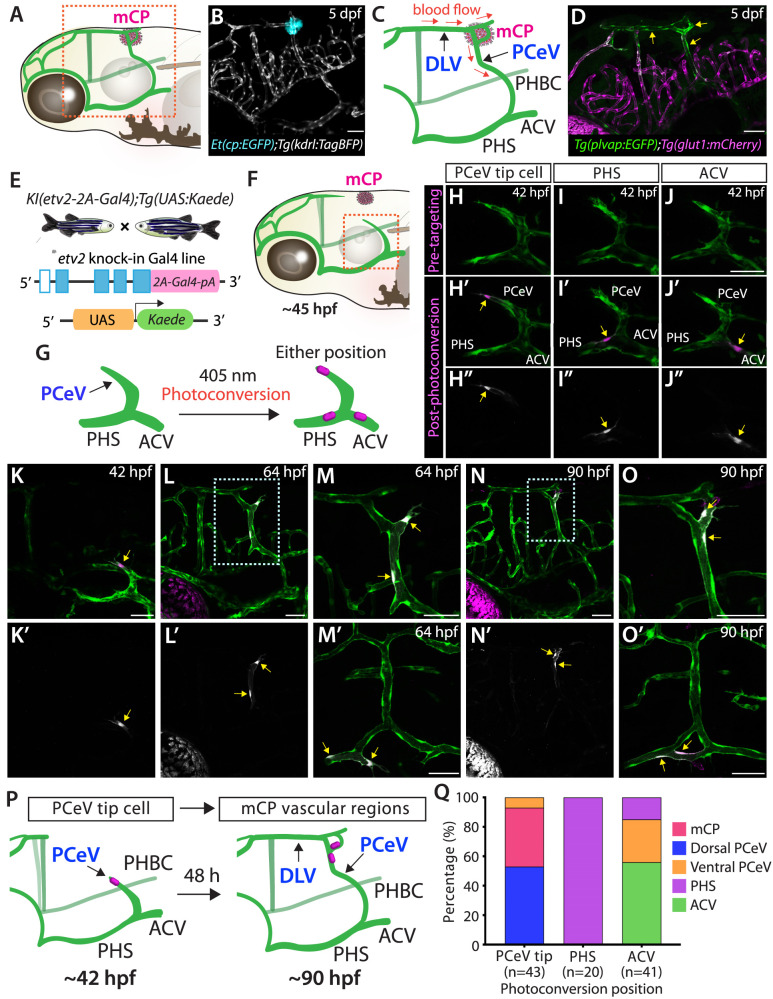
Single-cell-resolution fate mapping identifies PCeV tip cells as a major contributor to fenestrated mCP vasculature. ***A***, Schematic lateral view of the larval zebrafish head at ∼5 dpf, indicating the location of the myelencephalic choroid plexus (mCP) and its associated blood vessels. ***B***, Lateral view of a 5 dpf *Et(cp:EGFP);Tg(kdrl:TagBFP)* larval head, marking mCP epithelial cells (cyan) and blood vessels (white). ***C***, Schematic lateral view of larval zebrafish mCP vascular networks at ∼5 dpf, highlighting the dorsal longitudinal vein (DLV) and posterior cerebral vein (PCeV), two major vessels that form mCP vasculature along with their blood flow directions. ***D***, Lateral view of a 5 dpf *Tg(plvap:EGFP);Tg(glut1:mCherry)* larval head, showing strong expression of the fenestration marker *plvap* in the DLV, PCeV, and mCP vasculature (arrows). ***E***, Experimental setup for photoconversion experiments throughout this study. The knock-in line *KI(etv2-2A-Gal4)* carrying the *Tg(UAS:kaede)* transgenes were incrossed, and their heterozygous knock-in progeny were used. ***F***, Schematic lateral view of the embryonic zebrafish head at ∼45 hpf. ***G***, Magnified view of sprouting PCeV from the boxed area (***F***), showing the primary head sinus (PHS) and the anterior cardinal vein (ACV). ***H–J″***, Examples of photoconversion-induced, single-cell targeting of the PCeV tip cell (***H****–**H″***), PHS (***I****–**I″***), and ACV (***J****–**J″***) in 42 hpf *KI(etv2-2A-Gal4);Tg(UAS:Kaede)* embryos. Pre-photoconversion images are shown in (***H–J***), post-photoconversion in (***H*′**–***J*′**), and the photoconverted single endothelial cell (EC) at each location in (***H″***–***J″***). Arrows point to photoconverted ECs. ***K***–***O*′**, Photoconversion performed at 42 hpf (***K***, ***K*′**), with subsequent tracking at 64 (***L***-***M*′**) and 90 (***N***-***O*′**) hpf. Photoconverted PCeV tip cell (arrow in ***K***, ***K*′**) migrated to form mCP vascular networks (arrows, ***L***-***O*′**). Magnified views of the boxed area in ***L*** and ***N*** are shown in ***M*** and ***O***, respectively. Dorsal view images at 64 and 90 hpf are shown in ***M*′** and ***O*′**, respectively. ***P***, Diagrams illustrating the locations of photoconverted cells, as observed in experiments (***K–O*′**). ***Q***, Summary of photoconversion experiments targeting the PCeV tip cell, PHS, and ACV at 42 hpf. The locations of photoconverted ECs were examined at 64 hpf (*n* = 43 cells from PCeV tip cells, *n* = 20 from the PHS, and *n* = 41 from the ACV). Only PCeV tip cells contributed to mCP vascularization. Scale bars: 50 µm in ***B***, in ***J*** for ***H****–**J″***, in ***K*** for ***K*′**, in ***L*** for ***L*′**, in ***N*** for ***N*′**, in ***M***, ***M*′**, ***O***, ***O*′**; 100 µm in ***D***.

Across vertebrates, the CPs serve to maintain brain homeostasis by producing CSF and clearing metabolic wastes (Jeong et al., 2024). In mammals, CPs develop on the surfaces of the lateral, third, and fourth brain ventricles (Liddelow, 2015), but their deep anatomical locations and in utero embryonic development present significant technical challenges for live and longitudinal imaging. To overcome this limitation and investigate embryonic developmental processes of the CPs, we leveraged the zebrafish model. The zebrafish CPs form in two anatomical locations, referred to as the diencephalic and myelencephalic CPs (dCP and mCP), which are equivalent to the third and fourth ventricular CPs in mammals, respectively (Bill and Korzh, 2014). Both the dCP and mCP are located near the dorsal brain surfaces, enabling non-invasive visualization of CP morphogenesis. The mCP is larger in size and positioned in the hindbrain visualized by the *Et(cp:EGFP)* enhancer trap line that specifically labels CP epithelial cells (Fig. 1*A*,*B*; Henson et al., 2014).

Previous studies showed that mCP vasculature develops at the junction between two blood vessels, the dorsal longitudinal vein (DLV), and bilateral posterior cerebral veins (PCeV; Bill et al., 2008; Garcia-Lecea et al., 2008; Henson et al., 2014). Under physiological conditions, the DLV exclusively supplies blood to the mCP, which in turn drains through the bilateral PCeVs (Fig. 1*C*; Bill et al., 2008; Garcia-Lecea et al., 2008; Henson et al., 2014). The mCP vasculature exhibits molecular signatures characteristic of fenestrated vessels, including high expression of the fenestration-associated protein PLVAP and low expression of the BBB marker GLUT1 (Fig. 1*D*).

Prior work indicates that these two vessels arise from distinct cranial vascular domains and that the PCeV may sprout from the bilateral primary head sinus (PHS), a major embryonic head vein (Isogai et al., 2001). However, its precise origin and early developmental processes have yet to be characterized. To delineate mCP vascular lineages, we established an EC-specific photoconversion approach using the *KI(etv2-2A-Gal4)* knock-in line, which drives Gal4 expression under the endogenous promoter of the early angioblast marker *etv2* (Chestnut and Sumanas, 2020). This line was then crossed with *Tg(UAS:Kaede)* adults to produce progeny expressing the photoconvertible Kaede protein specifically in ECs (Fig. 1*E*).

We achieved photoconversion of Kaede from green to red fluorescence in targeted ECs using 405 nm laser illumination for 1–2 s (Fig. 1*F*,*G*). This approach enabled single-cell labeling of ECs (Fig. 1*H–J″*), allowing their longitudinal tracking and fate mapping during development. To determine the PCeV’s contributions to mCP vasculature, we initially targeted three vessel locations immediately after its sprouting at ∼42 h post fertilization (hpf), including the leading tip cell and neighboring ECs within the PHS or anterior cardinal vein (ACV; Fig. 1*H–J″*). By examining targeted ECs at 64 hpf, we found that PCeV tip cells consistently migrated to occupy mCP vascular regions (Fig. 1*K–M′*,*Q*; Fig. S1*A–C’*), whereas photoconverted ECs in the PHS or ACV mostly remained within their original territories (Fig. 1*Q*; Fig. S1*H–M*). At 90 hpf, photoconverted ECs were largely confined to the posterior side of the mCP vasculature by exhibiting only slight positional shifts from 64 hpf (Fig. 1*N–P*; Fig. S1*D–G*), indicating that these locations represent their final destinations. These experiments pinpoint PCeV tip cells as a specific EC population contributing to mCP vasculature.

### The PCeV arises from a junctional vessel that connects two major veins of the embryonic head

**Figure 2.**
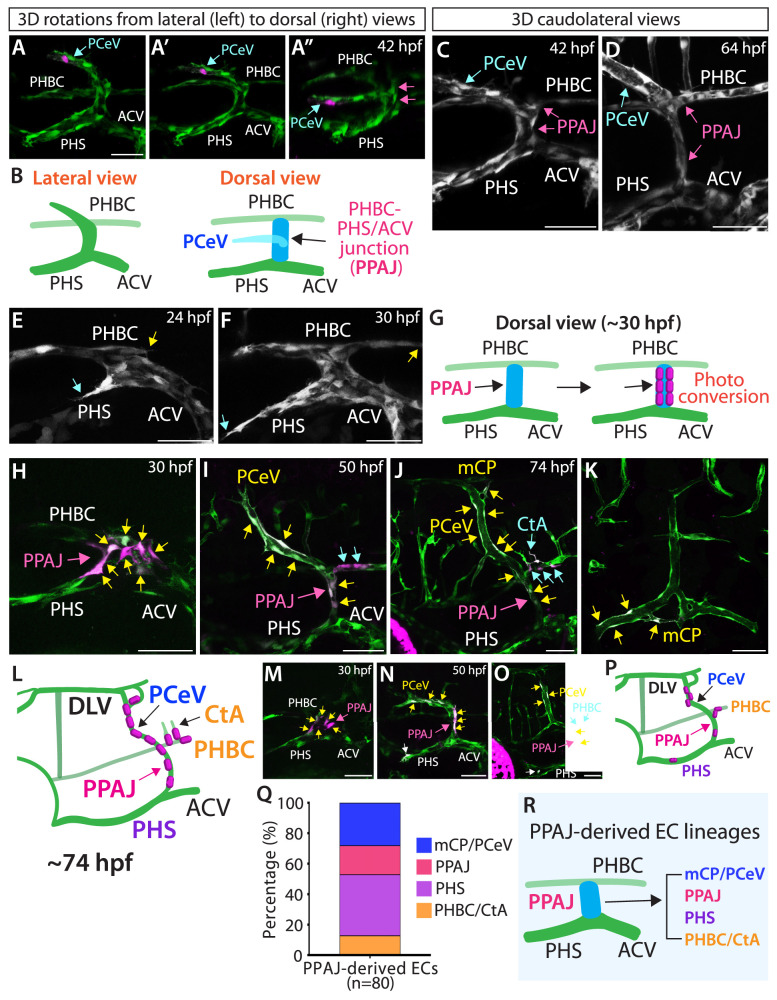
The PCeV arises from a junctional vessel that connects two major veins of the embryonic head. ***A–A***″, 3D-reconstructed images after photoconversion targeting the PCeV tip cell (cyan arrows) at 42 hpf. Lateral-to-dorsal rotational views revealed a junctional vessel that bridges the primordial hindbrain channel (PHBC) and the PHS/ACV (pink arrows, ***A″***). ***B***, Schematic lateral and dorsal views of the PCeV sprouting region at 42 hpf, illustrating a junctional vessel connecting the PHBC and the PHS/ACV, hereafter referred to as the PHBC–PHS/ACV junction (PPAJ). ***C***, ***D***, Caudolateral views of 3D-reconstructed *KI(etv2-2A-Gal4);Tg(UAS:Kaede)* head vasculature at 42 (***C***) and 64 (***D***) hpf, showing the PCeV (cyan arrows) originating from the PPAJ (pink arrows). ***E***, ***F***, Magnified images of *Tg(kdrl:EGFP)* embryos at 24 (***E***) and 30 (***F***) hpf, showing the primitive PPAJ at early developmental stages. Between these stages, anterior extension of the PHS (cyan arrows) and posterior formation of the PHBC (yellow arrows) were observed. PCeV sprouting has not yet been initiated by 30 hpf. ***G***, Experimental setup for photoconversion experiments (***H–Q***). ***H–K***, Photoconversion performed at 30 hpf (***H***), with subsequent tracking at 50 (***I***) and 74 (***J***, ***K***) hpf. Photoconverted ECs in the PPAJ (yellow arrows, ***H***) contributed to the PCeV and mCP vasculature (yellow arrows, ***I–K***), while also migrating into the PHBC that gave rise to the central artery (CtA; cyan arrows, ***I***, ***J***). Dorsal view at 74 hpf is shown in ***K***, presenting photoconverted ECs localized to the PCeV and the posterior mCP vasculature (yellow arrows). ***L***, Diagram illustrating the locations of photoconverted PPAJ cells/lineages at 74 hpf from experiments (***H–K***). ***M–O***, Photoconversion performed at 30 hpf (***M***), with subsequent tracking at 50 (***N***) and 74 (***O***) hpf. In this larva, photoconverted ECs in the PPAJ (yellow arrows, ***M***) contributed to the PCeV/mCP vasculature (yellow arrows, ***N***, ***O***), the PHBC (cyan arrows,*** O***), and the PHS (white arrow, ***N***, ***O***). ***P***, Diagram illustrating the locations of photoconverted PPAJ cells/lineages at 74 hpf from experiments (***M–O***). ***Q***, Quantification of photoconversion experiments targeting the PPAJ at 30 hpf. Four major EC locations were identified: the PCeV/mCP vasculature, PPAJ, PHBC/CtA, and PHS. The locations of photoconverted ECs were examined at 50 hpf, with a total of 80 cells PPAJ-derived cells identified and presented in the graph. ***R***, Summary of PPAJ photoconversion at 30 hpf, indicating EC lineages derived from the PPAJ. Scale bars: 50 µm in ***A*** for ***A****–**A″***, in ***C–F***, in ***H–K***, in ***M–O***.

While characterizing PCeV formation using photoconversion, we noticed that PCeV sprouting does not directly arise from the PHS/ACV, although it appears so in lateral views. To determine its precise sprouting site, we performed 3D reconstruction of confocal *z*-stack images (Fig. 2*A*,*B*). Visualizing sprouting PCeV from multiple angles revealed a junctional vessel that connects the PHS/ACV and the primordial hindbrain channel (PHBC), a major vein along the hindbrain (Fig. 2*A″*,*B*). Hereafter, we refer to this PHBC–PHS/ACV junction as the PPAJ, as it has no previously assigned name.

Further caudolateral views of 3D-reconstructed images from different developmental stages revealed the PPAJ as a precise initiation site of PCeV sprouting (Fig. 2*C*,*D*). Since PPAJ development has not been well characterized, we next examined this process at 24 and 30 hpf. At 24 hpf, the primitive PPAJ connecting the PHBC and ACV was already evident, while the PHS and the posterior PHBC segment relative to the PPAJ had not yet formed (Fig. 2*E*). By 30 hpf, the PPAJ had narrowed, with the PHS and PHBC extending anteriorly and posteriorly, respectively, whereas PCeV sprouting had not been initiated (Fig. 2*F*). To test whether the PPAJ serves as an embryonic source of ECs contributing to mCP vasculature, we photoconverted multiple ECs within this structure at 30 hpf (Fig. 2*G*). By examining these photoconverted ECs at 50 and 74 hpf, we found that they migrated to form mCP vasculature in a manner similar to PCeV tip cells targeted at ∼42 hpf (Figs. 2*H–L*, 1*K–P*). As observed for the PHS and ACV, ECs photoconverted in the PHBC at 42 hpf largely stayed within their original domains (Fig. S2). Collectively, these fate-mapping experiments demonstrate that the PPAJ and PCeV tip cells share EC lineages giving rise to fenestrated mCP vasculature.

### Developmental fate mapping indicates the presence of specialized embryonic sources that contribute ECs to both fenestrated mCP vasculature and BBB-type vessels

The PCeV and PPAJ fate-mapping experiments allowed us to identify notable differences: PCeV tip cell targeting exclusively gives rise to mCP vasculature and the dorsal portion of the PCeV, whereas PPAJ targeting resulted in more diverse EC contributions, including the PCeV/mCP vasculature (28%), the PHS (40%), the PHBC (13%), and PPAJ itself (19%; Fig. 2*H–R*; Fig. S3). Intriguingly, the PHBC gave rise to the central artery (CtA), a vessel that later forms the BBB, indicating that the embryonic PPAJ is a specialized vascular region that contributes ECs to both fenestrated mCP vasculature and BBB-associated vessels.

**Figure 3.**
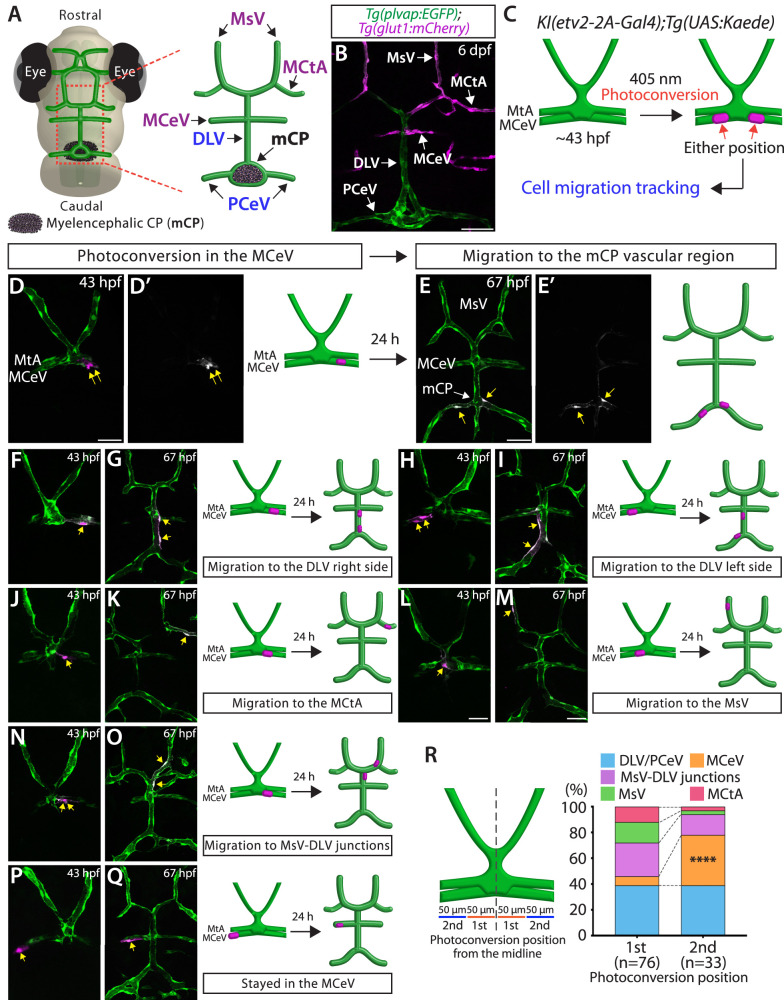
Developmental fate mapping pinpoints a specific embryonic MCeV segment as another major contributor to fenestrated mCP vasculature. ***A***, Schematic dorsal view of the larval zebrafish head at 6 dpf, indicating the location of the mCP and its associated blood vessels: DLV, PCeV, mesencephalic vein (MsV), middle cerebral vein (MCeV), and mesencephalic central artery (MCtA). ***B***, Dorsal view of a 6 dpf *Tg(plvap:EGFP);Tg(glut1:mCherry)* larval head, showing strong expression of the BBB marker *glut1* in the MsV, MCeV, and MCtA, and restricted *plvap* expression in the DLV and PCeV. ***C***, Experimental setup for photoconversion experiments (***D–R***). ***D–E*′**, Photoconversion performed in the MCeV at 43 hpf (arrows, ***D***, ***D*′**), with subsequent tracking at 67 hpf (***E***, ***E*′**). The photoconverted MCeV cells migrated to form the mCP vasculature (yellow arrows). ***F–Q***, Examples of photoconverted ECs in the MCeV that contributed to distinct cranial vessels. Photoconversion was performed at 43 hpf (***F***, ***H***, ***J***, ***L***, ***N***, ***P***) and subsequently tracked at 67 hpf (***G***, ***I***, ***K***, ***M***, ***O***, ***Q***). Photoconverted ECs (arrows) were localized to the DLV (***G***, ***I***), MCtA (***K***), MsV (***M***), MsV–DLV junctions (***O***), or remained in the MCeV (***Q***). ***R***, Summary of photoconversion experiments targeting the MCeV at 43 hpf, showing diverse migration locations of ECs. Initial photoconversion positions were categorized based on their distance from the midline, as illustrated. Graphs include data from the 1st position (*n* = 76) and the 2nd position (*n* = 33). **** indicates *p* < 0.0001 by Fisher’s exact test. Scale bars: 50 µm in ***B***, in ***D*** for ***D*′**, in ***E*** for ***E*′**, in ***L*** for ***F***, ***H***, ***J***, ***N***, ***P***, in ***M*** for ***G***, ***I***, ***K***, ***O***, ***Q***.

Next, we investigated the DLV’s contributions to mCP vascularization (Fig. 3*A*,*B*). Previous work suggested that the DLV sprouts from the middle cerebral vein (MCeV) using a pan-EC transgenic line (Bill et al., 2008), but its limited resolution prevented from identifying specific ECs contributing to the mCP vasculature. To address this limitation, we employed our photoconversion-based, single-cell labeling approach to target ECs on the MCeV at ∼43 hpf prior to the initiation of DLV sprouting and track them 24 h later at 67 hpf. At 43 hpf, the MCeV forms in close proximity to the mesencephalic artery (MtA) at the midbrain–hindbrain boundary, with the MCeV residing caudally (Fig. 3*C*). As DLV sprouting progresses, the MCeV and MtA become intermingled, eventually appearing as a single vessel that is designated as the MCeV (Fig. 3*A*,*B*).

By targeting ECs in different segments of the MCeV, we were able to narrow down to a specific region that contributes ECs to the mCP vasculature (Fig. 3*D*,*E′*). This region is located near the dorsal midline junction (DMJ), the site where DLV sprouting initiates (Bill et al., 2008). Photoconversion of corresponding segments on both sides of the MCeV revealed that left-side cells mostly remained on the left, while right-side cells largely stayed on the right (Fig. 3*F–R*). When migrating into the mCP vascular region, photoconverted ECs predominantly localized to the anterior part of the mCP vasculature (Fig. 3*E*,*G*,*I*).

While repeating experiments to characterize these MCeV segments, we unexpectedly observed that ECs from this region also migrated to diverse vessels, including some that later acquired BBB molecular signatures, as indicated by strong GLUT1 expression (Fig. 3*B*). Our careful tracking of photoconverted ECs revealed five distinct destinations: DLV/PCeV (mCP vessels; Fig. 3*D–I*), mesencephalic central artery (MCtA; Fig. 3*J*,*K*), mesencephalic vein (MsV; Fig. 3*L*,*M*), MsV–DLV junctions (Fig. 3*N*,*O*), and MCeV (Fig. 3*P*,*Q*). To quantify these findings, we categorized photoconverted ECs based on their initial positions relative to the midline. We found that ECs located within 50 µm from the midline (1st position) most preferentially migrated to mCP vessels (39%; Fig. 3*R*), with the second most frequent destination being the MsV–DLV junctions (26%; Fig. 3*R*). Together, nearly 65% of cells from this position migrated to blood vessels with fenestrated features, though MsV–DLV junctions often displayed BBB marker expression on one side (Fig. 3*B*). The remaining 35% migrated to the MsV (16%), MCtA (12%), and MCeV (7%; Fig. 3*R*), the three vessels that display BBB characteristics.

Further analysis of ECs located 50–100 µm from the midline (2nd position) showed a markedly higher proportion of ECs remaining on the MCeV (39%; Fig. 3*R*). Accordingly, the overall proportions of ECs migrating to other vessels decreased, while migration to mCP vessels remained relatively high at 39%. Collectively, these characterizations reveal specific MCeV segments that contribute to the DLV and mCP vasculature. Together with our observations of PPAJ-derived ECs contributing to diverse vessels, these results suggest existence of embryonic vascular domains that generate both fenestrated and BBB EC subtypes.

### Time-lapse cell tracking identifies the embryonic MCeV as a specialized vessel that contributes ECs to multiple cranial vessels

**Figure 4.**
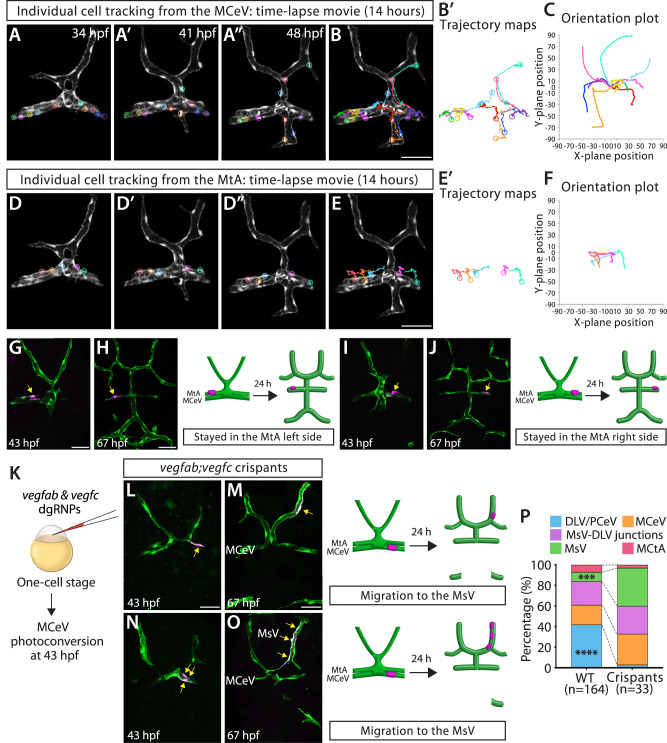
Time-lapse cell tracking identifies the embryonic MCeV as a specialized vessel that contributes ECs to multiple cranial vessels. ***A–A****″*, Dorsal view time-lapse images of *Tg(kdrl:EGFP)* cranial vasculature acquired between 34 and 48 hpf (14 h total). Images at 34 (***A***), 41 (***A*′**), and 48 (***A″***) hpf are shown. Ten ECs in the MCeV, each marked with a distinct colored circle, were tracked at 8 min intervals throughout the time lapse. ***B***, ***B*′**, Migration trajectories of the 10 tracked MCeV ECs at 48 hpf. Circles indicate their final positions. Trajectory traces alone are shown in (***B*′**). ***C***, Orientation plots of the 10 tracked MCeV ECs at 48 hpf present their individual displacement trajectories during the recorded period. These migration trajectories showed diverse orientations, with some cells exhibiting long-range movements. ***D****–**D″***, Dorsal view time-lapse images of the same *Tg(kdrl:EGFP)* embryo shown in ***A****–**A″***. Five ECs in the MtA, each marked with a distinct colored circle, were tracked at 8 min intervals for comparison with MCeV ECs. Images at 34 (***D***), 41 (***D*′**), and 48 (***D″***) hpf are shown. ***E***, ***E*′**, Migration trajectories of the five tracked MtA ECs at 48 hpf. Circles indicate their final positions. Trajectory traces alone are shown in (***E*′**). ***F***, Orientation plots of the five tracked MtA ECs at 48 hpf show their individual displacement trajectories during the recorded period. As compared with MCeV cells, MtA ECs exhibited restricted migration trajectories and limited movement directionality. ***G–J***, Tracking of photoconverted cells in the MtA from 43 (***G***, ***I***) to 67 hpf (***H***, ***J***). A photoconverted cell in the left MtA remained in the same area (arrow, ***G***, ***H***), whereas a photoconverted cell in the right MtA stayed on the right side (arrow, ***I***, ***J***). ***K***, Experimental setup for microinjection experiments (**L**-**P**). ***L–O***, Tracking of photoconverted MCeV cells in *vegfab;vegfc* crispants from 43 (***L***, ***N***) to 67 hpf (***M***, ***O***). These crispants lacked the DLV, and photoconverted ECs migrated to the MsV (arrows, ***M***, ***O***). ***P***, Graphs comparing all combined photoconversion results between WT (*n* = 164) and *vegfab;vegfc* crispants (*n* = 33). As compared with WT animals, *vegfab;vegfc* crispants exhibited markedly reduced contributions to the DLV/PCeV and significantly increased contributions to the MsV. These graphs include similar proportions of ECs photoconverted from the 1st position (62% for WT and 61% for *vegfab;vegfc* crispants). *** and **** indicate *p* < 0.001 and *p* < 0.0001, respectively, by Fisher’s exact test. Scale bars: 50 µm in ***B*** for ***A****–**A″***, in ***E*** for ***D****–**D″***, in ***G*** for ***I***, in ***H*** for ***J***, in ***L*** for ***N***, in ***M*** for ***O***.

To investigate whether the embryonic MCeV serves as a specific source of ECs for the mCP and nearby BBB-type vessels, we next tracked individual EC behaviors in the MCeV and compared them with those in the adjacent MtA. To this end, we analyzed serial time-lapse images collected every 8 min from 34 to 48 hpf, a developmental time window when DLV sprouting begins and extends. Comparing their migration trajectories revealed diverse migratory patterns of MCeV-derived ECs (Fig. 4*A–C*; Movie 1), consistent with our photoconversion-based lineage tracing results. In contrast, ECs in the MtA exhibited neither long-range movements nor diverse trajectories, instead remaining within the MtA (Fig. 4*D–F*; Movie 2). Photoconversion of ECs in the MtA confirmed this observation (Fig. 4*G–J*), with nearly all marked cells staying in the MtA (13 out of 14 cells; the remaining one migrated to the MCtA). Time-lapse imaging following photoconversion enabled clear visualization of MCeV ECs migrating to diverse vessels, including the DLV (Fig. S4, Movie 3). Notably, none of the photoconverted ECs (*n* = 19) underwent cell divisions during the recorded time window, indicating that directed EC migration is a primary mechanism driving angiogenesis from this specialized region. Together, these results suggest that the early embryonic MCeV is a specialized source of ECs contributing to multiple cranial vessels, including the DLV and mCP vasculature.

**Movie 1.** Time-lapse cell tracking of 10 ECs in the embryonic MCeV. Time-lapse imaging of *Tg(kdrl:EGFP)* embryonic cranial vasculature from 34 to 48 hpf, showing 10 MCeV ECs tracked at 8 min intervals, with each cell marked by a distinct colored circle. [View online]

**Movie 2.** Time-lapse cell tracking of five ECs in the embryonic MtA. Time-lapse imaging of *Tg(kdrl:EGFP)* embryonic cranial vasculature from 34 to 48 hpf, showing five MtA ECs tracked at 8 min intervals, with each cell marked by a distinct colored circle. [View online]

**Movie 3.** Time-lapse tracking of photoconverted MCeV EC migration to the DLV. Time-lapse tracking of a photoconverted MCeV cell in a *KI(etv2-2A-Gal4);Tg(UAS:Kaede)* embryo from 45 to 55 hpf at 10 min intervals, showing its migration trajectory to the DLV. [View online]

### The combined loss of Vegfab and Vegfc causes DLV formation defects, resulting in a biased EC migration from the MCeV toward BBB-type vessels

Our recent studies showed that the combined loss of Vegfab and either Vegfc or Vegfd (or both) substantially increased DLV-absent phenotypes (Parab et al., 2021). To investigate how the deletion of these factors affects EC behaviors, we employed a CRISPR-based approach that enables efficient biallelic gene inactivation in F0 zebrafish (Quick et al., 2021). Specifically, we performed simultaneous injections of three distinct dual-guide CRISPR RNA/Cas9 ribonucleoproteins (dgRNPs) per gene, which achieved highly consistent generations of F0 knock-outs (crispants; Quick et al., 2021). By coinjecting a cocktail of three dgRNPs targeting *vegfab* and three targeting *vegfc* at the one-cell stage (Fig. 4*K*), we successfully recapitulated the DLV-absent phenotypes observed in stable *vegfab;vegfc* double mutants (Parab et al., 2021). Next, we injected this dgRNP cocktail into one-cell stage embryos carrying both the *KI(etv2-2A-Gal4)* and *Tg(UAS:Kaede)* transgenes to perform photoconversion. To align with the earlier wild-type (WT) experiments, we targeted equivalent MCeV segments in crispants and tracked photoconverted cells over the same time course. Notably, crispants showed a sharp reduction in cells migrating to mCP vessels and a dramatic increase in cells migrating to the MsV (Fig. 4*L–P*). These results suggest that Vegfab and Vegfc co-deletion caused dramatic shifts in EC migration locations from mCP vasculature to BBB-associated vessels.

### Embryonic venous lineages characterized by high *flt4* expression give rise to mCP vasculature

To identify specific Vegfrs contributing to mCP vascularization, we next examined *vegfr* gene expression using RNAscope in situ hybridization (Wang et al., 2012). This method generates specific punctate signals representing individual mRNA transcripts (Wang et al., 2012), enabling spatial mapping of gene expression at single-cell resolution with quantifiable data.

**Figure 5.**
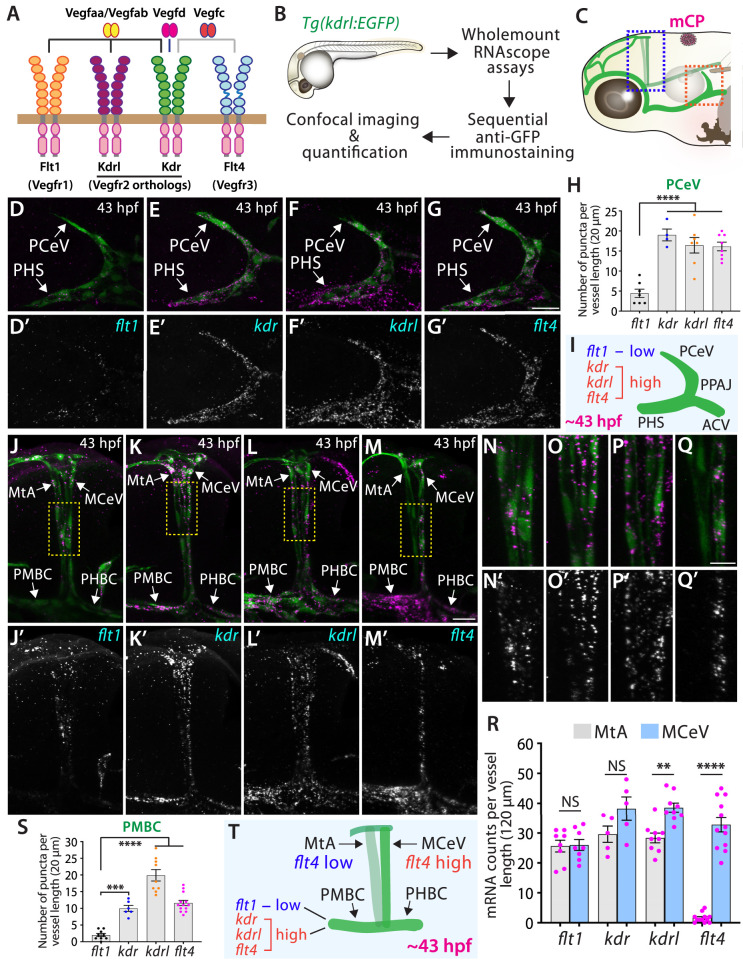
Embryonic venous lineages characterized by high *flt4* expression give rise to mCP vasculature. ***A***, Predicted receptor binding partners for zebrafish Vegfaa, Vegfab, Vegfc, and Vegfd. ***B***, Experimental workflow for sequential wholemount RNAscope and immunostaining, with subsequent imaging and quantification. ***C***, Schematic lateral view of the embryonic zebrafish head at ∼43 hpf. Boxed areas indicate the two vascular regions analyzed for gene expression. ***D–G*′**, RNAscope assays in 43 hpf *Tg(kdrl:EGFP)* embryos using *flt1* (***D***, ***D*′**), *kdr* (***E***, ***E*′**), *kdrl* (***F***, ***F*′**), and *flt4* (***G***, ***G*′**) probes. The head region indicated by the orange boxed area in ***C*** were imaged. RNAscope signals alone are shown in (***D*′**–***G*′**). ***H***, Quantification of mRNA signals with the indicated probes in the sprouting PCeV at 43 hpf. ***I***, Diagram summarizing the relative *vegfr* expression levels (***D–G*′**) observed in this vascular region at 43 hpf. ***J–M*′**, RNAscope assays in 43 hpf *Tg(kdrl:EGFP)* embryos using *flt1* (***J***, ***J*′**), *kdr* (***K***, ***K*′**), *kdrl* (***L***, ***L*′**), and *flt4* (***M***, ***M*′**) probes. The head region indicated by the blue boxed area in ***C*** were imaged. RNAscope signals alone are shown in ***J*′**–***M*′**. ***N–Q*′**, Magnified images from the boxed areas (***J–M***), showing *flt1* (***N***, ***N*′**), *kdr* (***O***, ***O*′**), *kdrl* (***P***, ***P*′**), and *flt4* (***Q***, ***Q*′**) expression in the MtA and MCeV. ***R***, Quantification of mRNA signals with the indicated probes in the MtA and MCeV at 43 hpf. ***S***, Quantification of mRNA signals with the indicated probes in the primordial midbrain channel (PMBC) at 43 hpf. ***T***, Diagram summarizing the relative *vegfr* expression levels (***J–M*′**) observed in this vascular region at 43 hpf. In panels ***H***, ***R***, and ***S***, values represent means ± SEM (**, ***, and **** indicate *p* < 0.01, *p* < 0.001, and *p* < 0.0001, respectively, by one-way ANOVA followed by Tukey's post hoc test). Scale bars: 20 µm in ***G*** for ***D–G*′**, in ***M*** for ***J–M*′**; 10 µm in ***Q*** for ***N–Q*′**.

In zebrafish, the Vegfr family includes four members: Flt1, Kdr, Kdrl, and Flt4 (Fig. 5*A*; Vogrin et al., 2019). Biochemical studies showed that Vegfc binds to Flt4 and Kdr, whereas Vegfd binds exclusively to Kdr (Vogrin et al., 2019). Vegfa paralogs (Vegfaa and Vegfab) interact with Kdr and Kdrl (VEGFR2 orthologs in zebrafish; Bahary et al., 2007), and Flt1 acts as a decoy receptor modulating Vegfa signaling (Matsuoka et al., 2016).

To assess *vegfr* expression during early cerebrovascular development, we performed RNAscope assays at 43 hpf using EC-specific *Tg(kdrl:EGFP)* embryos (Fig. 5*B*). At this stage, two cranial locations were analyzed from lateral views: the PCeV and DLV sprouting regions (Fig. 5*C*). In the PCeV sprouting region, the PCeV displayed strong expression of *kdr*, *kdrl*, and *flt4* (Fig. 5*E–G′*). Similar expression levels of these genes were observed in the PHS and PPAJ, whereas the arterial marker *flt1* showed markedly lower expression in all these vessels (Fig. 5*D–I*; Gurung et al., 2022).

In the DLV sprouting region, the MCeV, which gives rise to the mCP vasculature, exhibited clear expression of all four Vegf receptors (Fig. 5*J–R*). Notably, *flt4* expression was largely restricted to the MCeV, with nearly absent expression in the adjacent MtA (Fig. 5*M*,*M′*,*Q–R*). Previous work indicates that the MCeV originates from the primordial midbrain channel (PMBC) and forms at the junction of the PMBC and PHBC (Ulrich et al., 2011). We observed that both the PMBC and PHBC displayed low levels of *flt1* expression (Fig. 5*J*,*J′*,*S*), whereas *kdr*, *kdrl*, and *flt4* were strongly expressed (Fig. 5*K–M′*,*S*), supporting the venous identity of these vessels and the MCeV. Collectively, these expression analyses provide evidence that both the DLV and PCeV arise from the embryonic venous lineages characterized by strong *flt4* expression (Fig. 5*I*,*T*).

### *flt4* serves as a specific marker distinguishing developing mCP vasculature from neighboring BBB-type vessels

**Figure 6.**
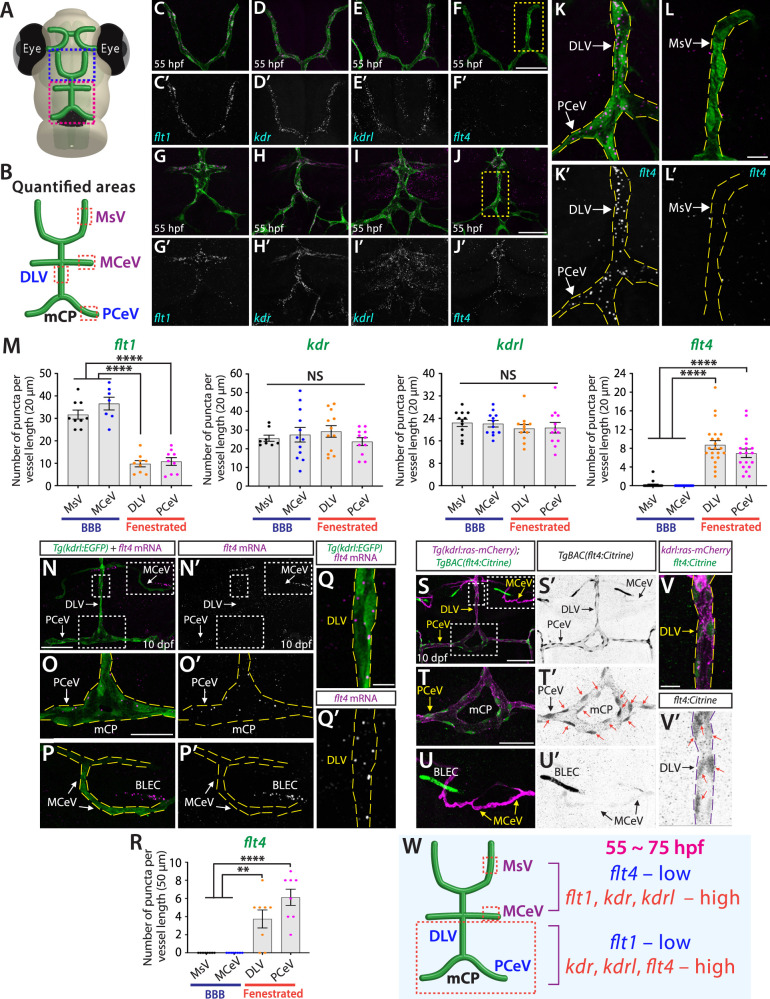
*flt4* acts as a specific marker distinguishing developing mCP vasculature from neighboring BBB-type vessels. ***A***, Dorsal view schematic of the embryonic zebrafish head at ∼55 hpf, showing the mCP location and imaged vascular regions. ***B***, Vascular diagram illustrating the approximate vessel locations used for quantifications. ***C–L*′**, RNAscope assays in 55 hpf *Tg(kdrl:EGFP)* embryos using *flt1* (***C***, ***G***), *kdr* (***D***, ***H***), *kdrl* (***E***, ***I***), and *flt4* (***F***, ***J***) probes. Panels ***C–F*′** show the MsV (blue box in ***A***), and panels ***G–J*′** show the DLV, PCeV, and MCeV (pink box in ***A***). RNAscope signals alone are shown in ***C′–F′*** and ***G′–J′***. ***K***, ***K*′**, Magnified images from the boxed area (***J***), showing high *flt4* expression in the DLV and PCeV. ***L***, ***L*′**, Magnified images from the boxed area (***F***), showing absent *flt4* expression in the MsV. ***M***, Quantification of mRNA signals per vessel in 55 hpf *Tg(kdrl:EGFP)* embryos for the indicated probes. ***N–Q′***, RNAscope assays in 10 dpf *Tg(kdrl:EGFP)* larvae using the *flt4* probe. Magnified views of the mCP (***O***, ***O′***), MCeV (***P***, ***P′***), and DLV (***Q***, ***Q′***) regions are shown, corresponding to the boxed areas in ***N***, ***N′***. ***R***, Quantification of *flt4* mRNA signals per vessel at 10 dpf. ***S***, ***S′***, Dorsal views of a 10 dpf *TgBAC(flt4:Citrine);Tg(kdrl:ras-mCherry)* larval head, showing *flt4* reporter expression in the DLV and PCeV forming mCP vasculature. ***T–V′***, Magnified views of the mCP (***T***, ***T′***), MCeV (***U***, ***U′***), and DLV (***V***, ***V′***) regions are shown, corresponding to the boxed areas in ***S***. Robust *flt4* expression was detected in the DLV, PCeV, and mCP vasculature (red arrows, ***T′***, ***V′***), but not in the MCeV (arrows, ***U′***). ***W***, Diagram summarizing the relative *vegfr* expression levels observed across distinct cranial vessels at 55 and 75 hpf. Refer to Figure S5 for detailed expression results at 75 hpf. In panels ***M***, ***R***, values represent means ± SEM (** and **** indicate *p* < 0.01 and *p* < 0.0001, respectively, by one-way ANOVA followed by Tukey’s post hoc test). Scale bars: 50 µm in ***F*** for ***C–F′***, in ***J*** for ***G–J′***, in ***N*** for ***N′***, in ***S*** for ***S′***; 10 µm in ***L*** for ***K–L′***; 25 µm in ***O*** for ***O–P′***, in ***T*** for ***T–U′***; 10 µm in ***Q*** for ***Q′***, in ***V*** for ***V′***.

We next examined *vegfr* expression from dorsal views at 55 and 75 hpf, developmental stages during which active DLV sprouting and mCP vascularization occur. To compare gene expression levels between vessels, we measured mRNA signals per vessel length (Fig. 6*A*,*B*). At 55 hpf, *kdr* and *kdrl* were uniformly expressed across cranial vessels (Fig. 6*D*,*E′*,*H*,*I′*,*M*). In contrast, *flt4* expression was restricted to the DLV and PCeV, notably in both tip and stalk cells (Fig. 6*F–F′*,*J–L′*,*M*), with nearly absent expression in the MsV and MCeV at this stage. Conversely, *flt1* expression was more prominent in the MsV and MCeV, while significantly lower expression was still detected in the DLV and PCeV (Fig. 6*C–C′*,*G–G′*,*M*). These overall expression patterns persisted at 75 hpf (Fig. S5). Together, these results show that all *vegfr* genes are expressed in developing mCP vessels and identify *flt4* as a specific early marker distinguishing mCP vessels from neighboring BBB-type vasculature.

Our previous expression analysis indicated that the three key Vegf ligands (Vegfab, Vegfc, and Vegfd) required for mCP vascularization are derived from multiple cellular sources, including meningeal fibroblasts, mCP epithelial cells, and the sprouting DLV (Parab et al., 2021). However, that study did not include detailed analyses of their expression near the PPAJ. Thus, we examined their expression patterns using our previously validated BAC transgenic Gal4FF reporters crossed with *Tg(UAS:EGFP-CAAX)* fish, which allows visualization of Vegf ligand-expressing cells by membrane-bound EGFP. At 55 hpf, expression analysis revealed prominent *vegfc* reporter expression in close proximity to the PPAJ, PHS, and ACV (Fig. S6*A,C,C’*). In contrast, *vegfab* reporter expression was more restricted to the rostral side of the PPAJ (Fig. S6*B,B’*), while the *vegfd* reporter did not label this region (Fig. S6*D,D’*). These results suggest that paracrine actions of Vegfab and Vegfc drive PCeV sprouting through their putative receptors expressed in ECs of the PPAJ.

### Heterogeneous *flt4* expression across the cerebrovasculature persists at later developmental stages

To determine whether the differential *flt4* expression patterns observed between mCP and BBB-type vessels persist after mCP vascularization, we examined *flt4* expression at 10 dpf using both RNAscope and the established *flt4* BAC reporter line *TgBAC(flt4:Citrine)* (van Impel et al., 2014). This BAC reporter strongly labeled lymphatic-like ECs in the primitive meninx during larval stages, which were previously identified as brain lymphatic EC (BLEC; van Lessen et al., 2017). Consistent with the RNAscope results at 10 dpf (Fig. 6*N–O′*,*Q*,*Q′*), clear *flt4* reporter activity was detected in the DLV, PCeV, and mCP vasculature at this stage (Fig. 6*S*,*T′*,*V*,*V′*), whereas only faint or nearly absent signals were observed in the MCeV and MsV (Fig. 6*P*,*P′*,*R*,*U*,*U′*). These data further support *flt4*’s persistent and specific expression in mCP vessels across the cerebrovasculature (Fig. 6*W*) and suggest that Flt4 signaling contributes to the development of heterogeneous vascular networks in this brain region.

### Co-requirements of Vegfr2 and Vegfr3 signaling in mCP vascularization

**Figure 7.**
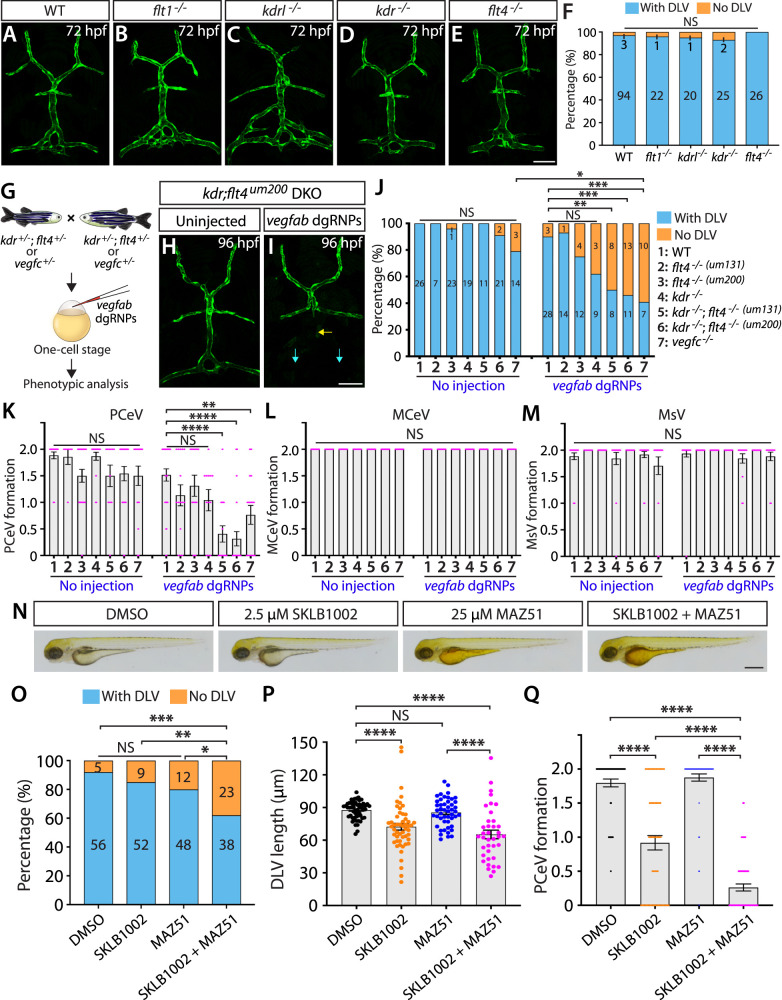
Genetic or pharmacological co-inhibition of Vegfr2 and Vegfr3 signaling exacerbates mCP vascularization defects. ***A–E***, Dorsal views of 72 hpf WT (***A***), *flt1^−/−^* (***B***), *kdrl^−/−^* (***C***), *kdr^−/−^* (***D***), and *flt4^−/−^* (***E***) cranial vasculature visualized by the *Tg(kdrl:*EGFP*)* transgene. Although the *kdrl^−/−^* larva displayed an apparently enlarged and more branched mCP vascular network, all the single knock-outs formed the DLV and PCeV. ***F***, Percentage of larvae with and without the DLV for each genotype (number of animals examined per genotype is listed). ***G***, Experimental workflow for microinjection experiments (***H–M***). ***H***, ***I***, Dorsal views of 96 hpf *kdr^−/−^;flt4^−/−^* (um200) larval vasculature visualized by the *Tg(kdrl:*EGFP*)* transgene after no injection (***H***) or *vegfab* dgRNP injections (***I***). Injected mutants lacked both the DLV (yellow arrow) and PCeV (cyan arrows) (***I***). ***J***, Percentage of 96 hpf larvae of indicated genotypes and treatments with and without the DLV (number of animals examined per genotype and treatment is listed). ***K–M***, Quantification of PCeV (***K***), MCeV (***L***), and MsV (***M***) formation in uninjected and *vegfab* dgRNPs injected larvae at 96 hpf. ***N***, Bright-field images of 72 hpf larvae treated from 34 to 72 hpf with DMSO (vehicle), 2.5 μM SKLB1002, 25 μM MAZ51, or a combination of both inhibitors. ***O***, Percentage of the 72 hpf *Tg(kdrl:EGFP)* larvae with and without the DLV after the indicated treatments (number of animals examined per treatment is listed). ***P***, DLV length quantification in 72 hpf *Tg(kdrl:EGFP)* larvae after the indicated treatments. ***Q***, Quantification of PCeV formation in 72 hpf *Tg(kdrl:EGFP)* larvae after the indicated treatments. In panels ***K–M***, ***P***, ***Q***, values represent means ± SEM (** and **** indicate *p* < 0.01 and *p* < 0.0001, respectively, by one-way ANOVA followed by Tukey’s post hoc test). In panels ***J***, ***O***, *, **, and *** indicate *p* < 0.05, *p* < 0.01, and *p* < 0.001, respectively, by Fisher’s exact test. Scale bars: 50 µm in ***E*** for ***A–E***, in ***I*** for ***H***; 500 µm in ***N***.

Based on the expression of all *vegfrs* in developing mCP vessels, we next investigated the contribution of each receptor to mCP vascularization using their respective null mutants (Fig. 7*A–F*). Consistent with previous reports, the genetic loss of *kdrl* severely disrupted brain angiogenesis, including CtA formation in the brain parenchyma (Fetsko et al., 2022; Schevenels et al., 2024). Despite this severe defect, mCP vasculature still formed (Fig. 7*C*,*F*), suggesting that Kdrl signaling is differentially required for the development of BBB-type and mCP vessels. Similarly, other *vegfr* knock-outs formed both the DLV and PCeV, with no apparent defects in mCP vascularization.

Next, we examined the combined roles of these receptors, given their overlapping expression patterns and the reported genetic interactions during vascular development (Covassin et al., 2006; Bahary et al., 2007; Vogrin et al., 2019). To efficiently screen potential Vegfr combinations, we used the streamlined F0 knock-out approach described earlier (Quick et al., 2021). By analyzing *kdr*, *kdrl*, and *flt4* knock-outs in all possible combinations, we found that the combined loss of *flt4* and either *kdr* or *kdrl* did not result in mCP vascularization defects (data not shown), while deletions of both *kdr* and *kdrl* caused global embryonic vascular defects, as previously reported (Covassin et al., 2006; Bahary et al., 2007). Despite these severe angiogenic disruptions, we frequently observed DLV-like vessel formation (data not shown), suggesting that these Vegfr2 orthologs are not the only receptors required for DLV formation.

To explore a potential role for Flt4 in this process, we induced the genetic loss of *kdr* and *flt4* in *vegfab* knock-outs. This strategy was expected to mitigate global angiogenic defects caused by *kdrl* deletion, while reducing this receptor activation induced by Vegfab, a major angiogenic ligand crucial for mCP vascularization (Parab et al., 2021). To validate this approach, we first injected *vegfab* dgRNPs into progeny from *vegfc^+/−^* incrosses and analyzed phenotypes at 96 hpf (Fig. 7*G*), given that Vegfc can signal through Flt4 and Kdr (Vogrin et al., 2019) and that *vegfab^−/−^;vegfc^−/−^* larvae exhibit significantly enhanced mCP vascularization defects (Parab et al., 2021). In *vegfc^−/−^* larvae injected with *vegfab* dgRNPs, we observed a significant increase in DLV-loss phenotypes compared with uninjected *vegfc^−/−^* and injected *vegfc^+/+^* animals (Fig. 7*J*), recapitulating the co-requirement of Vegfab and Vegfc for DLV formation (Fig. 4*K–O*; Parab et al., 2021).

Using this approach, we next assessed mCP vascularization in single and double mutants of *kdr* and *flt4* with and without *vegfab* dgRNP injections. While *vegfab* dgRNP-injected *kdr* and *flt4* single mutants did not exhibit significantly increased defects in DLV and PCeV formation, injected *kdr;flt4* double mutants displayed vascular defects comparable to those observed in injected *vegfc^−/−^* larvae (Fig. 7*J*,*K*). Notably, these injected *kdr;flt4* double mutants showed no defects in MsV and MCeV formation (Fig. 7*L*,*M*), demonstrating the vessel specificity of the observed phenotypes. These findings suggest a co-requirement of Kdr, Flt4, and Kdrl in mCP vascularization.

### Genetic mutants lacking the Flt4 cytoplasmic domain phenocopy mCP vascularization defects observed in *flt4* null mutants

To further investigate Flt4 function in mCP vascularization, we utilized the additional mutant allele *flt4^um200^*, which lacks tyrosine residues in the cytoplasmic domain necessary for activating PI3K and MEK/ERK pathways (Shin et al., 2016). This mutant allowed us to assess the role of Flt4 receptor function and its potential effector pathways in this process.

Under the same experimental settings described above, *flt4^um200^* mutants displayed mCP vascularization defects consistent with those observed in the null *flt4^um131^* allele. Specifically, *flt4^um200^* single mutants alone did not show significant defects in DLV and PCeV formation, regardless of *vegfab* dgRNP injections. Conversely, *kdr;flt4^um200^* double mutants injected with *vegfab* dgRNP displayed severe mCP vascularization defects (Fig. 7*H–I*), similar to those observed in injected *kdr;flt4^um131^* double mutants or *vegfc^−/−^* larvae (Fig. 7*J–M*). These findings indicate that Vegfc/Flt4-dependent PI3K and MEK signaling is critical for mCP vascularization.

### Chemical co-inhibition of Vegfr2 and Flt4 signaling exacerbates mCP vascularization defects

To overcome the limitations of the *kdr;kdrl* double mutant analysis that led to global angiogenic defects, we employed a pharmacological approach. Specifically, we used SKLB1002 and MAZ51 as Vegfr2- and Flt4-specific inhibitors, respectively, and treated embryos between 34 and 72 hpf. SKLB1002 treatment inhibited DLV extension in a dose-dependent manner (Fig. S7*A–I*; Fig. 7*P*) and impaired PCeV formation without causing overt morphological abnormalities (Fig. 7*N*,*Q*). However, SKLB1002 alone did not significantly increase DLV-absent phenotypes, even at elevated doses (Fig. 7*O*,Fig. S7*H*). Conversely, MAZ51 treatment alone caused a slight increase in DLV-absent phenotypes (Fig. 7*O*) but did not significantly affect DLV extension or PCeV formation (Fig. 7*P*,*Q*). Notably, co-treatment with both inhibitors exacerbated DLV-absent phenotypes and PCeV formation defects (Fig. 7*N–Q*), further suggesting a co-requirement of Vegfr2 and Flt4 signaling in these processes. The additional loss of *flt1* had no effect on these defects (Fig. S7*J–M*), ruling out Flt1 receptor signaling in this process. Collectively, both genetic and pharmacological data support a model in which Vegfr2 and Flt4 act cooperatively to promote mCP vascularization.

### PI3K and MEK signaling pathways are both necessary for mCP vascularization

VEGF ligands and receptors regulate angiogenesis by activating downstream kinases, including PI3K, ERK, and PLCK (Simons et al., 2016). To investigate which intracellular signaling pathways in ECs are required for mCP vascularization downstream of Vegfr activation, we employed a pharmacological approach to inhibit individual pathways within a defined time window.

**Figure 8.**
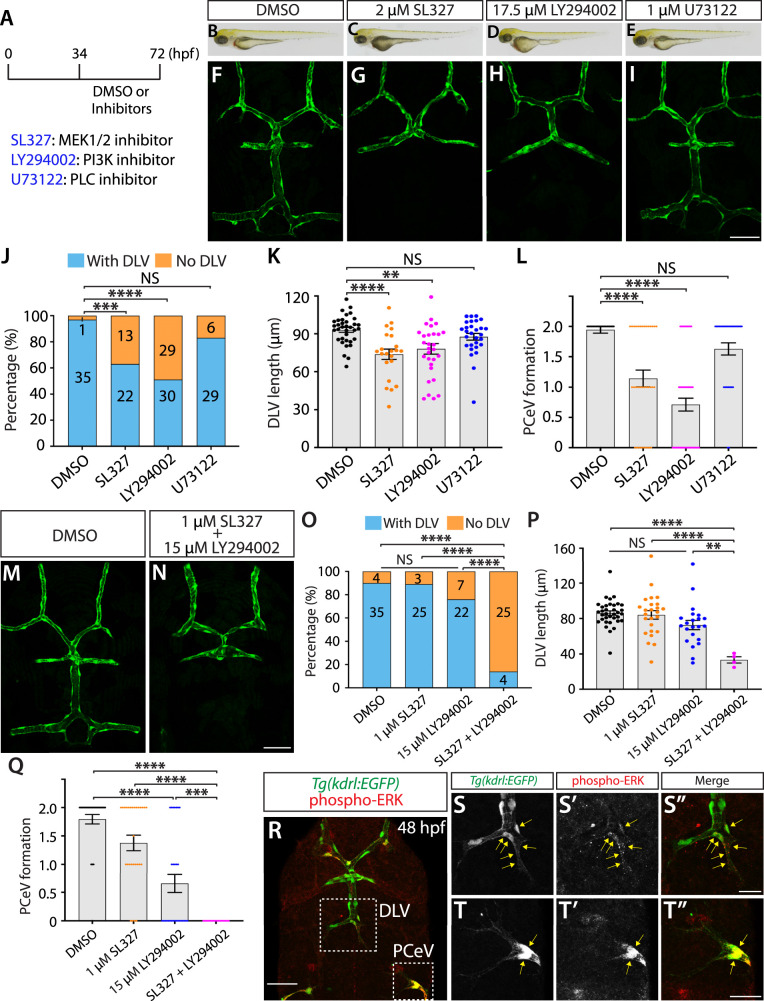
Chemical inhibition of the MEK and PI3K pathways disrupts mCP vascularization. ***A***, Experimental time course for chemical treatments ***B–L*** and ***M–Q***. ***B–E***, Bright-field images of 72 hpf larvae after treatment with DMSO (***B***), 2 µM SL327 (***C***), 17.5 µM LY294002 (***D***), or 1 µM U73122 (***E***). ***F–I***, Dorsal views of 72 hpf *Tg(kdrl:EGFP)* larval vasculature after treatment with DMSO (***F***), 2 µM SL327 (***G***), 17.5 µM LY294002 (***H***), or 1 µM U73122 (***I***). The larvae treated with SL327 or LY294002 lacked both the DLV and PCeV (***G***, ***H***). ***J***, Percentage of 72 hpf *Tg(kdrl:EGFP)* fish with and without the DLV after the indicated treatments (number of animals examined per treatment is listed). ***K***, DLV length quantification in 72 hpf *Tg(kdrl:EGFP)* larvae that formed the DLV after the indicated treatments (*n* = 35 for DMSO, *n* = 22 for SL327, *n* = 30 for LY294002, and *n* = 29 for U73122). ***L***, Quantification of PCeV formation in 72 hpf *Tg(kdrl:*EGFP) larvae after the indicated treatments (*n* = 36 for DMSO, *n* = 35 for SL327, *n* = 59 for LY294002, and *n* = 35 for U73122). ***M***, ***N***, 72 hpf *Tg(kdrl:EGFP)* larval cranial vasculature after treatment with DMSO (***M***) or a combined solution of 1 µM SL327 and 15 µM LY294002 (***N***). The fish subjected to the combined treatment lacked both the DLV and PCeV (***N***). ***O***, Percentage of 72 hpf *Tg(kdrl:EGFP)* larvae with and without the DLV after the indicated treatments (number of animals examined per treatment is listed). ***P***, DLV length quantification in 72 hpf *Tg(kdrl:EGFP)* larvae that formed the DLV after the indicated treatments (*n* = 35 for DMSO, *n* = 25 for 1 µM SL327, *n* = 22 for 15 µM LY294002, and *n* = 4 for combined SL327/LY294002). ***Q***, Quantification of PCeV formation in 72 hpf *Tg(kdrl:*EGFP) larvae after the indicated treatments (*n* = 39 for DMSO, *n* = 28 for 1 µM SL327, *n* = 29 for 15 µM LY294002, and *n* = 29 for combined SL327/LY294002). ***R***, 48 hpf *Tg(kdrl:EGFP)* embryos immunostained for EGFP and phosphorylated ERK. Magnified images of the DLV and PCeV (boxed areas in ***R***) are shown in ***S****–**S″*** and ***T****–**T″***, respectively. Arrows indicate phosphorylated ERK immunoreactivity in tip cells of both vessels. In panels ***K***, ***L***, ***P***, ***Q***, values represent mean ± SEM (**, ***, and **** indicate *p* < 0.01, *p* < 0.001, and *p* < 0.0001, respectively, by one-way ANOVA followed by Tukey’s post hoc test). In panels ***J***, ***O***, *** and **** indicate *p* < 0.001 and *p* < 0.0001, respectively, by Fisher’s exact test. Scale bars: 50 µm in ***I*** for ***F–I***, in ***N*** for ***M***, in ***R***; 20 µm in ***S″*** for ***S****–**S″***, in ***T″*** for ***T****–**T″***.

Specifically, we treated embryos between 34 and 72 hpf with the following chemicals: SL327, a MEK1/2 inhibitor (Favata et al., 1998); LY294002, a selective PI3K inhibitor (Gharbi et al., 2007); and U73122, a PLCK inhibitor (Smith et al., 1990). We first determined sublethal doses of each inhibitor that caused minimal gross morphological changes (Fig. 8*A–E*). Treatment with 2 μM SL327 or 17.5 μM LY294002 significantly increased the DLV-absent phenotype (nearly 40 and 50%, respectively; Fig. 8*F–H*,*J*), shortened DLV lengths (Fig. 8*K*), and impaired PCeV formation (Fig. 8*L*). In contrast, 1 μM U73122 treatment did not cause any of these vessel formation defects (Fig. 8*I–L*). These results indicate that the PI3K and MEK pathways, but not PLCK, are required for DLV and PCeV formation.

We next tested how inhibition of both the PI3K and MEK pathways affects mCP vascularization by co-treating embryos with LY294002 and SL327. To this aim, we applied lower concentrations of each inhibitor (1 μM SL327 and 15 μM LY294002). At these dosages, individual treatments caused only mild effects on DLV formation, as judged by the incidence of DLV-absent phenotypes (Fig. 8*O*) and DLV length measurements (Fig. 8*P*). In contrast, this co-treatment substantially increased DLV formation defects (86% of larvae lacking the DLV) and severely disrupted PCeV formation (Fig. 8*M–Q*). Similar results were obtained with the half doses of the combined inhibitors (Fig. S8), suggesting that both the PI3K and MEK pathways are necessary for mCP vascularization. Combined with our genetic findings using the *flt4^um200^* mutant allele (Fig. 7*H–K*), these pharmacological results indicate the PI3K and ERK pathways as important effectors of Vegfr2/Flt4-dependent mCP vascularization.

### Tip cells of the developing DLV and PCeV exhibit MEK/ERK pathway activation

If the PI3K and MEK pathways are required for mCP vascularization, their activation should be evident during DLV and PCeV formation. To test this idea, we performed wholemount immunostaining with an antibody specific to phosphorylated ERK (pERK), a key activation step in the MAPK signaling cascade. At 48 hpf, when both the DLV and PCeV were actively extending, we observed strong pERK signals in the leading tip cells of both vessels (Fig. 8*R–T″*). Despite the absence of a reliable antibody against phosphorylated AKT suitable for zebrafish, these results suggest that MEK/ERK signaling is active during mCP vascularization, consistent with our genetic and pharmacological findings.

## Discussion

**Figure 9.**
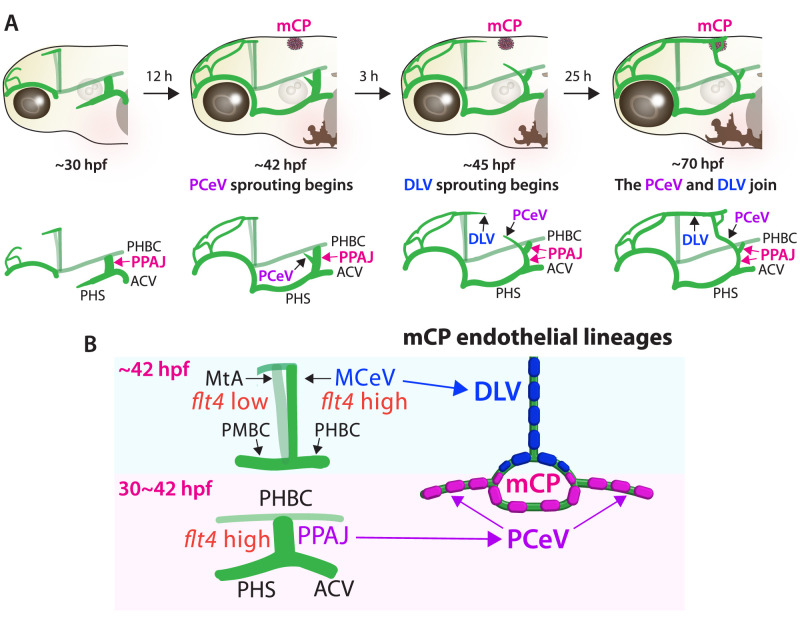
Stepwise development of mCP vascularization and endothelial cell lineage mapping in the fenestrated mCP vasculature. ***A***, Diagrams illustrating key developmental steps of mCP vascularization, highlighting two major vascular sources: the DLV and PCeV, which originate from the venous vessels MCeV and PPAJ, respectively. ***B***, Schematics illustrating endothelial lineage contributions from the DLV and PCeV to the mCP vasculature. Fate mapping indicates that PCeV-derived lineages predominantly populate the posterior side of mCP vasculature, whereas DLV-derived lineages mainly contribute to the anterior region. Both the DLV and PCeV arise from embryonic venous vessels characterized by strong *flt4* expression, indicating that the mCP vasculature is exclusively of venous origin.

In this study, we investigated embryonic endothelial origins of hindbrain fenestrated CP vasculature using the zebrafish model. CP vasculature significantly differs from BBB-type vessels in molecular, structural, and functional properties, yet its vascular lineages remained undefined. Using photoconversion-based EC lineage tracing and time-lapse cell tracking, we identified two embryonic vascular domains that give rise to the mCP vasculature. Two blood vessels, the DLV and PCeV, contribute to mCP vascularization, and they sprout from the embryonic MCeV and PPAJ, respectively (Fig. 9*A*). Early embryonic MCeV and PPAJ exhibit venous features characterized by strong *flt4* expression, suggesting that fenestrated mCP vasculature is exclusively of venous origin (Fig. 9*B*).

Our fate mapping and time-lapse imaging suggest the presence of specialized embryonic venous domains that contribute ECs to both fenestrated and BBB-characteristic vessels. These observations indicate that the early embryonic PPAJ and MCeV are heterogeneous structures with the capacity to generate ECs of distinct fates, analogous to the lymphatic niche in the cardinal vein (Nicenboim et al., 2015) and the hematopoietic niche within the aortic endothelium (Bertrand et al., 2010; Boisset et al., 2010; Kissa and Herbomel, 2010). How the embryonic PPAJ and MCeV acquire such heterogeneous properties remains an open question and requires further investigation. One possibility is that this process involves mechanisms similar to lymphatic-venous segregation, whereby mesenchyme-derived paracrine Vegfc signaling activates the transcription factor Prox1 in lymphatic ECs sprouting from the cardinal vein, which in turn enhances Flt4 expression (Srinivasan et al., 2014). Consistent with this idea, our BAC transgenic analyses reveal prominent *vegfc* expression in regions adjacent to the PPAJ (Fig. S6*C,C’*). In contrast, *vegfab* BAC reporter expression is more spatially restricted, marking a small subset of cells associated with the rostral PPAJ (Fig. S6*B,B’*). It is possible that these ligands act together to provide asymmetric instructive cues that promote rostral sprouting of the PCeV from the PPAJ. Subsequently, meningeal fibroblast-like cells exhibiting *vegfab*, *vegfc*, and/or *vegfd* BAC reporter expression, along with mCP epithelial cells displaying prominent *vegfab* expression (Fig. S6), may guide both the DLV and PCeV toward the mCP region. Systematic analysis of the spatiotemporal expression of *prox1* paralogs and *flt4* within the DMJ and PPAJ and their functional roles will help test this model.

In support of this proposed model, we identified highly restricted *flt4* expression across cerebrovascular subtypes during mCP vascularization: strong expression in both tip and stalk cells of the growing DLV and PCeV that give rise to fenestrated mCP vasculature, but nearly absent *flt4* expression in neighboring BBB-type vessels. This differential *flt4* expression distinguishes these cerebrovascular subtypes and persists at least until 10 dpf. Sustained *flt4* expression in mCP vasculature is in contrast with the transient *flt4* expression typically observed in tip cells during sprouting angiogenesis (Siekmann and Lawson, 2007; Tammela et al., 2008) and aligns with the previous study reporting VEGFR3 expression in fenestrated capillaries of the fetal and adult human CP (Partanen et al., 2000). In mature vasculature, Flt4 is typically recognized as a lymphatic EC-specific marker, along with Prox1, Lyve1, and other genes (Kaipainen et al., 1995; Banerji et al., 1999; Wigle and Oliver, 1999; Oliver, 2022). Given the emerging concept of hybrid vasculature expressing both blood and lymphatic identity markers (Aspelund et al., 2014; Park et al., 2014; Kenig-Kozlovsky et al., 2018; Pawlak et al., 2019; Schnabellehner et al., 2024), it will be interesting to examine lymphatic EC marker expression in CP capillaries and explore the upstream mechanisms driving selective *flt4* expression within brain capillaries in future studies.

To date, very few markers specific to fenestrated brain ECs have been identified. PLVAP has been widely used as a primary marker to distinguish fenestrated ECs from BBB-associated ones in mature vasculature (Hallmann et al., 1995; Stan et al., 2004; Parab et al., 2023). However, given the broad expression of PLVAP in immature ECs (Hallmann et al., 1995; Umans et al., 2017), our data suggest that *flt4* serves as a reliable early marker for identifying fenestrated brain ECs. Our genetic and pharmacological results further support Flt4’s functional role in regulating fenestrated CP vessel formation with little impact on neighboring BBB-type vessel development. The two distinct *flt4* mutant alleles we examined exhibited consistent angiogenic defects in mCP vascularization, providing evidence for a requirement of this receptor signaling in this process. Pharmacological treatments complement our genetic results, collectively suggesting that Vegfr2 and Flt4 cooperate in mCP vascularization and that the PI3K and ERK pathways are required for this process. A recent study indicates that Flt4 and Aplnrb signaling may genetically interact in PCeV formation (Herdt et al., 2025), supporting our genetic and pharmacological findings on Flt4 function.

In summary, our study presents the first detailed characterization of embryonic endothelial lineages that give rise to fenestrated CP vasculature in vertebrates. We propose a model in which Vegfr2/Flt4-dependent signaling acts as a crucial angiogenic pathway for CP vascularization, with Flt4 providing specific signals to allow the formation of CP vasculature segregated from BBB-type vessels.
